# The Role of Human Centromeric RNA in Chromosome Stability

**DOI:** 10.3389/fmolb.2021.642732

**Published:** 2021-03-31

**Authors:** Simon Leclerc, Katsumi Kitagawa

**Affiliations:** Greehey Children’s Cancer Research Institute, Department of Molecular Medicine, University of Texas Health Science Center at San Antonio, San Antonio, TX, United States

**Keywords:** cancer, cenRNA, lncRNA, kinetochore, centromere, chromosome instability

## Abstract

Chromosome instability is a hallmark of cancer and is caused by inaccurate segregation of chromosomes. One cellular structure used to avoid this fate is the kinetochore, which binds to the centromere on the chromosome. Human centromeres are poorly understood, since sequencing and analyzing repeated alpha-satellite DNA regions, which can span a few megabases at the centromere, are particularly difficult. However, recent analyses revealed that these regions are actively transcribed and that transcription levels are tightly regulated, unveiling a possible role of RNA at the centromere. In this short review, we focus on the recent discovery of the function of human centromeric RNA in the regulation and structure of the centromere, and discuss the consequences of dysregulation of centromeric RNA in cancer.

## Introduction

Accurate chromosome segregation is fundamental for cell division. Errors in this process can lead to chromosome instability, leading to aneuploidy, which is correlated with cancer (Zhu et al., 2011; Santaguida and Amon, 2015). The centromere is a component of each chromosome used for accurate chromosome segregation. The kinetochore, the structure responsible for binding the chromosome to spindle microtubules and for chromosome movement during cell division, is assembled on the centromere (Van Hooser et al., 2001). The identity and inheritance of the centromere are thought to be determined epigenetically by the deposition of the species-specific histone 3 variant CENH3 (CENP-A in mammals, CID in D. melanogaster, and Cse4 in *S. cerevisiae*) nucleosomes interspersed with classical Histone 3 nucleosomes (Blower et al., 2002; Fukagawa and Earnshaw, 2014; Niikura et al., 2016). The centromere of the budding yeast *S. cerevisiae* consists of a 100 nucleotides DNA sequence motif and is referred to as a point centromere (Pluta et al., 1995). In all other eukaryotes, centromeres are composed of repetitive DNA sequences on several hundred kilobases, referred to as regional centromeres (Pluta et al., 1995). Furthermore, the DNA composition of each centromere presents a high variation between each chromosome (Eichler, 1999; Melters et al., 2013). The “centromere paradox” refers to how highly diverse centromere sequences are, even in closely related eukaryotes (Eichler, 1999). Human centromeres are composed of α-satellite repeated tandemly to form a block of satellites, called higher-order repeat (HOR) that are composed of a set number of monomers that vary from 2 to 34 (Willard, 1985; Willard et al., 1986; Alexandrov et al., 1993; McNulty et al., 2017). Despite the repetitive sequences composing the centromere, this region is transcriptionally active, with the transcription of genes in rice (Nagaki et al., 2004; Mizuno et al., 2011). For other organisms, centromeric DNA encodes for siRNA (Grishok et al., 2000; Volpe et al., 2002; Zilberman et al., 2003; Fukagawa et al., 2004; Pal-Bhadra et al., 2004) and long-non-coding RNA called cenRNA (Wong et al., 2007; Carone et al., 2009).

## Transcription at the Centromere

A dynamic balance between euchromatin and heterochromatin at the centromeric region is required for active kinetochore (Nakano et al., 2008) and transcription of these regions (Saffery et al., 2003). In Drosophila and human cells, the centromeric region presents a distinct set of histone modification, one being the H3 Lys4-diMe, a modification associated with open but not active euchromatin, and lack clear marker for both heterochromatin (such as H3 Lys9 diMe) or euchromatin (such as H3 Lys9 Ac) (Sullivan and Karpen, 2004). Centromeric DNA encodes mainly non-coding RNA which are transcribed by the RNA polymerase II (Chan et al., 2012; Rošić et al., 2014), and may participate in the assembly and function of the centromeres.

In *S. cerevisiae*, transcription at the centromere is regulated in an RNA polymerase II-dependent manner, and the level of transcription is critical to maintaining the centromere function (Ohkuni and Kitagawa, 2011; Hildebrand and Biggins, 2016). Higher-level transcription at the centromere leads to its inactivation of chromosome missegregation (Hill and Bloom, 1987), while a base level of transcriptional activity is required for centromere function (Ohkuni and Kitagawa, 2011). This regulation is realized by a competition between the transcription factors Cbf1 and Ste12 and the silencing factors Sir1, Hst1, or Cdc14 (Ohkuni and Kitagawa, 2011). Point centromere presents in *S. pombe* display similar behavior, with too much or too little centromeric non-coding RNA leading to centromere malfunction (Ling and Yuen, 2019). Similarly, in mice, forced accumulation of the centromeric minor satellite transcripts leads to defects in chromosome segregation (Bouzinba-Segard et al., 2006), while in human cells, the inhibition of the RNA polymerase II during mitosis leads to a decrease in centromeric α satellite transcription, anaphase lagging cells and abnormal chromosome splitting (Chan et al., 2012; McNulty et al., 2017). This suggests that, in mammals, the transcription at the centromere is tightly regulated, maybe by its interaction with the nucleolus that may induce its repression (Bury et al., 2020). In human cells, one of the regulators of cenRNA transcription is ZFAT, which binds to a specific DNA sequence present on each centromere (Ishikura et al., 2020). Its presence at the centromere increases the centromeric level of the histone acetylase KAT2B and its product the histone 4 acetylated at the lysine 8 (Ishikura et al., 2020). This leads to the recruitment of the RNA pol II through BRD4 to the centromere, thus allowing ZFAT to control cenRNA transcription (Ishikura et al., 2020).

During mitosis, most regions within the condensed chromosomes are transcriptionally inactive, while centromeric regions are not (Chan et al., 2012; Liu et al., 2015). During the cell cycle, there is a stable centromeric transcription to ensure a stable centromere and kinetochore cohesion (Liu et al., 2015), but there is still some peak of expression occurring at different phases of the cell cycle. For example, in budding yeast, the centromeric expression occurs mainly in the S phase (Ling and Yuen, 2019), while in mice, a peak in the G2/M phase is observed (Ferri et al., 2009). In human cells, the RNA pol II is transcriptionally active at the mitotic kinetochore (Chan et al., 2012). R-loops, the byproduct of DNA-RNA hybridization, have important physiological functions but are also contributing to defects in genome integrity and chromosome fragility (Aguilera and García-Muse, 2012). In budding yeast, the accumulation of R-loops at the centromere is associated with a defect in kinetochore integrity (Mishra et al., 2021). In human, DHX9 is one element generating centromeric R-loops (Chakraborty et al., 2018), and while R-loops causes genomic instability in S phase (Buisson et al., 2015), they are necessary for faithful mitosis through a mitotic specific ATR pathway, independent of DNA damage or replication, but dependent of Aurora B and R-loops, ensuring accurate chromosome segregation (Aze et al., 2016; Kabeche et al., 2018).

A unique set of non-coding RNA is produced from each human alpha satellite array, and kinetochore assembly requires this RNA at the active centromere site (McNulty et al., 2017). Also, active transcription at the centromere is required for *de novo* deposition and incorporation of CENP-A into chromatin, and as such, to keep the centromere position (Choi et al., 2011; Bobkov et al., 2018). The products of transcription are highly variable lengths of RNAs, ranging from 500 to 2000+ nucleotides (Bouzinba-Segard et al., 2006; McNulty et al., 2017); in mice, these undergo post-transcriptional processing to generate smaller RNAs from 120 to 150 nucleotides (Bouzinba-Segard et al., 2006). These long non-coding RNAs are referred to as cenRNA.

## siRNA at the Centromere

It is thought that the RNAi has an important role in chromosome function since the suppression of different RNAi proteins displays a chromosome lagging phenotype in many eukaryotes (Gutbrod and Martienssen, 2020). In *Cryptococcus* yeast, loss of the RNAi machinery triggers the attrition of the retrotransposon composing the centromere, resulting in the shortening of centromere length (Yadav et al., 2018). In *S. Pombe*, transcription of the centromeric sequence and their processing by the RNAi machinery through the ribonuclease Dicer is required for chromosome segregation and gene silencing (Provost et al., 2002; Volpe et al., 2002). Rpb7, a subunit of RNA Pol II, actively recognizes a centromeric promoter and transcribes a part of the centromere to a pre-siRNA required for RNAi-directed chromatin silencing (Djupedal et al., 2005).

In higher eukaryotes, the effect of the RNAi machinery is less clear. The depletion of Dicer in mouse embryonic stem cells leads to an accumulation of centromeric transcripts (Kanellopoulou et al., 2005; Murchison et al., 2005). However, these two studies obtain opposite results about the loss of heterochromatin modification around the centromere, possibly caused by a small amount of truncated Dicer produced by an allele that may retain or inhibit the cytosine methylation at the centromeres (Murchison et al., 2005). Dicer processes the double-strand RNA derived from the centromeric repeat into smaller RNAs ranging from 25 to 150 nucleotides, that may be incorporated into the mammalian complex RITS (Kanellopoulou et al., 2005). In chicken-human hybrid cells, the depletion of Dicer results in the accumulation of transcript from α-satellite sequences, without affecting the localization of CENP-A or CENP-C, but results in the diffusion of HP1 throughout the entire chromosome, opposed to focusing on the centromere (Fukagawa et al., 2004). In *Drosophila* cells, the RNAi machinery affects the localization of HP1 and the heterochromatic silencing (Pal-Bhadra et al., 2004). More recently, Huang et al. showed that in human cell lines, the level and distribution of chromosome-associated α-satellite RNA on the centromere is dependent on the presence of siRNA generated by Dicer, guided by AGO2, and direct the deposition of CENPC at the centromere (Huang et al., 2015).

Multiple proteins interact with these RNAs, and are involved in heterochromatin formation or are centromere-specific factors, such as the H3K9 methyltransferase SUV39H1 and 2 (Johnson et al., 2017; Velazquez Camacho et al., 2017), HP1 (Maison et al., 2011), CENP-A (Quénet and Dalal, 2014), CENP-A chaperone protein HJURP (Quénet and Dalal, 2014) CENP-C (Huang et al., 2015) and Aurora B (Blower, 2016).

## RNA Is a Component of the Centromere and the Kinetochore

The fact that centromeres contain RNA has been known for several decades (Heidemann et al., 1977; Rieder, 1979). In maize, products of transcription of centromeric retrotransposons and satellite repeat—small RNAs that range from 40 to 200 nucleotides—are tightly bound to the centromeric histone 3, the maize equivalent of CENP-A, and components of the kinetochore (Topp et al., 2004). This transcriptional activity may facilitate the replacement and maintenance of the centromere (Topp et al., 2004). Maize CENP-C protein presents a DNA and an RNA binding motif, both of them required for the recruitment of CENP-C at the centromere, and that binding to RNA highly increases CENP-C affinity to DNA (Du et al., 2010). In Drosophila, the cenRNA produced from the 359-bp repeat satellite III is required for the localization of both CENP-A and CENP-C at the centromere for all chromosomes (Rošić et al., 2014). In human cells, cenRNA transcription is required for the localization of CENP-A and HJURP at the centromere (Quénet and Dalal, 2014). Depletion of CENP-A does not change the transcription level of these RNAs, but the reduction of cenRNA reduces the loading of CENP-A at the centromere, suggesting that the cenRNA themselves recruit CENP-A (McNulty et al., 2017). Besides, treatment by an RNAse results in the de-colocalization of CENP-C from mature human kinetochore, suggesting that cenRNA may also have a major function in the recruitment of CENP-C and its localization in the centromere (Wong et al., 2007).

In Xenopus eggs, the centromeric region is transcribed as cenRNA, and this cenRNA localizes to mitotic centromeres, chromatin, and spindle, through the chromosome passenger complex (CPC) allowing the activation of Aurora B (Blower, 2016). The transcription of these RNAs at the centromere region is required for attachment of the kinetochores to the mitotic spindle (Blower, 2016). Centromeric minor satellite RNAs from mice are also associated with proteins of the CPC, Aurora B, and Survivin (Ferri et al., 2009). Also, the kinase activity of Aurora B is dependent on RNA, and these satellite RNAs may be required (Ferri et al., 2009). In humans, α satellite RNA associates with Aurora B and INCENP, another component of the CPC, and their depletion from the cell result in the mislocalization of the CPC from the centromere region (Ideue et al., 2014).

There is some evidence of direct interaction between cenRNAs and centromeric protein, pointing out the critical roles of cenRNAs in multiple organisms. In Drosophila, the cenRNA produced from the 359-bp repeat satellite III binds to CENP-C (Rošić et al., 2014), while in human cells, cenRNAs are associated with CENP-A and CENP-C at the centromere (McNulty et al., 2017). Still, in human cells, CENP-C directly interacts *in vitro* with ∝ satellite RNAs through an RNA binding motif (Wong et al., 2007). In mammals, cenRNA interaction with Aurora B may play an important role in recruiting the CPC to the centromere (Ferri et al., 2009; Ideue et al., 2014).

**FIGURE 1:**
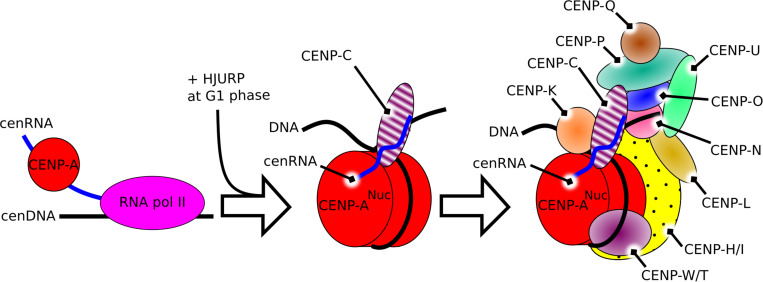
Schematic representation of the constitutive role of cenRNA in the structure of the centromere in humans. *Left*, the product of transcription of alpha-satellite DNA by the RNA polymerase II recruits CENP-A. *Middle*, after the constitution of the CENP-A nucleosome, cenRNA recruits CENP-C. *Right*, during mitosis, cenRNA may still be associated with the CCAN, and recruit the CPC via interaction with Aurora B to build the whole kinetochore.

Overall, cenRNA, as a component of the kinetochore, is involved in the recruitment of CENP-A, its chaperone HJURP, and CENP-C at the centromere, and is directly interacting with CENP-A and CENP-C. These components are involved in the recruitment of other parts of the centromere constitutive associated-network (CCAN) (Hori et al., 2008; Klare et al., 2015). Together with Aurora B, these components build the full kinetochore (Figure 1).

## Cancer Relevance

In pancreatic and other epithelial human cancers, overexpression of α-satellite transcripts (cenRNA) (Ting et al., 2011) is correlated with a poor clinical prognosis (Zhang et al., 2016). One function of BRCA1 is to facilitate the mono-ubiquitination of histones H2A associated with satellite DNA, which suppresses their transcription (Zhu et al., 2011). In some types of breast cancer, a lack of BRCA1 function leads to an overexpression of satellite RNA (Zhu et al., 2018). The over-expression of these RNAs destabilize the DNA replication forks, which results in the formation of R-loop and DNA damage at the centromere region (Reddy et al., 2011; Crossley et al., 2019), which is sufficient to induce aneuploidy (Zhu et al., 2018). Overexpression of alpha-satellite transcripts has been identified in both mouse and human pancreatic adenocarcinomas, within average a 40-fold increase compared to normal tissue (Ting et al., 2011). It is thought to be caused by the alteration of heterochromatin silencing, affecting both satellite DNA and LINE retrotransposons; the latter is linked with the expression of neuroendocrine genes proximal to LINE sequence (Ting et al., 2011). In general, high levels of cenRNA promote high CIN that correlates with tumor metastasis (Zhu et al., 2011; Turajlic and Swanton, 2016; Chan et al., 2017). CIN creates micronuclei caused by errors in chromosome segregation, resulting in the presence of cytosol DNA that activates the cGas-STING pathway (cyclic GMP-AMP synthase stimulator of interferon gene) (Bakhoum et al., 2018). Suppression of CIN reduces metastasis even in highly aneuploid cells while activating CIN promotes cellular invasion and metastasis (Bakhoum et al., 2018). A prolonged cell arrest before mitosis, caused by CIN, reduces the inflammatory signaling and the anti-tumor immunity in a cGas dependent manner (Bakhoum et al., 2018; Chen et al., 2020). Overexpression of Mad2 increases the karyotypes complexity through a CIN effect in Kras tumors, and if in the first step, the growth of the tumor is disadvantageous, in the second step a selective pressure on oncogene elimination leads to the development of persistent subclones that grow steadily (Rowald et al., 2016; Sheltzer et al., 2017). Similarly, in spontaneous lymphomas and lung tumors, an increased rate of CIN drives an elevated level of tumorigenesis, however, in the case of induced tumor formation, an increased rate of CIN is a more effective inhibitor than activator of tumorigenesis (Weaver et al., 2007).

**FIGURE 2:**
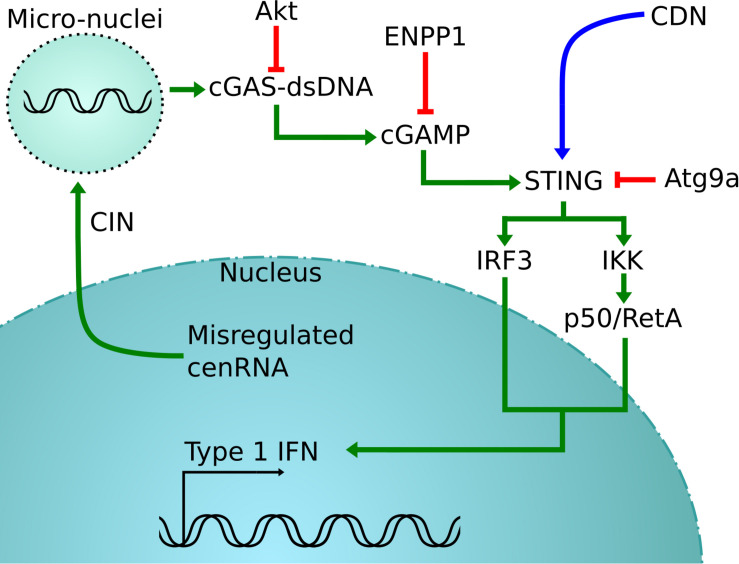
cGas-STING activation pathway. In green is the normal pathway, where cytosolic DNA created by the misregulation of cenRNA is detected by cGAS, which will cause the activation of STING (Corrales et al., 2015; Jiang et al., 2020). This is detrimental for cancer cells that will inactive STING using different strategies, in red (Saitoh et al., 2009; Xiong et al., 2018). One possible rescue is to inject synthetic CDN to force the activation of STING, shown in blue (Corrales et al., 2015).

Thus, detection of cenRNA might be a good predictor of cancer prognosis. In support of this concept, detection of pericentromeric SatII RNA, satellite RNAs present at specific heterochromatin located at the edges of the centromere, in biopsies is more tightly correlated with detection than the standard method (Ting et al., 2011; Bersani et al., 2015; Kishikawa et al., 2016). Sensing cytosol DNA to measure the amount of CIN also seems promising (Corrales et al., 2015; Jiang et al., 2020). Clinical trials are testing agonists of STING-nucleotide, with direct microinjection in the tumor of a synthetic cyclic dinucleotide (CDN) derivative that activates all human STING alleles (Corrales et al., 2015; Flood et al., 2019). Since CIN produces cytosol DNA that is detected by cGas-STING, this results in the activation of the STING pathway, involving the production of IFN-β and other cytokines (Ishikawa and Barber, 2008; Figure 2).

## Conclusion

Centromeres are essential for chromosome segregation, and while this function is preserved across species, their structure and mechanisms underlying presents large differences. However, one common particularity is that the centromere DNA sequence is actively transcribed. The resulting RNA is then processed to result in RNAi in yeast and in mammals or cenRNA in mammals. These cenRNAs have been shown to be an indispensable part of the centromere by interacting with CENP-A and CENP-C, as well as actively recruiting other parts of the kinetochore. The dysregulation of the cenRNA expression leads to chromosome instability, which can result in some cases in metastasis of a cancer. One possible treatment to counter this effect is the injection of a synthetic CDN that activates the cGas-STING pathway, able to detect the presence of cytosolic DNA resulting from CIN and activating an immune-response. One point to further investigate is with which mechanism higher eukaryotes can finely tune the expression of cenRNAs, and if a drug can avoid the overexpression of cenRNA in cancer cells.

## Author Contributions

SL wrote the manuscript. KK directed how to write the manuscript and edited it. Both authors contributed to the article and approved the submitted version.

## Conflict of Interest

The authors declare that the research was conducted in the absence of any commercial or financial relationships that could be construed as a potential conflict of interest.
